# Supplementary material for: CHROMATIX: computing the functional landscape of many-body chromatin interactions in transcriptionally active loci from deconvolved single cells
Source: Genome Biol. 2020 Jan 16;21:13. doi: 10.1186/s13059-019-1904-z (PMC6966897; doi:10.1186/s13059-019-1904-z)
Supplement: Supplementary file 5 — Additional file 5 Review history. [file 13059_2019_1904_MOESM5_ESM.docx]

Review History

**First round of review**

**Reviewer 1**

**Were you able to assess all statistics in the manuscript, including the appropriateness of statistical tests used?**

No

**Were you able to directly test the methods?**

No

**Comments to author:**

Hi-C has provided significant insight into genome organization. However, due to its population nature, extracting information on the simultaneous interaction between multiple segments is difficult. Many body interactions are particularly interesting in light of recent developments that highlighted the importance of phase separation, which is inherently a many-body phenomenon, in genome organization.

The authors proposed a novel method for detecting many-body spatial interactions between genomic segments using polymer modeling. They further connect with epigenetic modifications using a machine learning approach to provide insight into molecular determinants for such many-body interactions. Overall, I find this paper interesting and support its publication. I do have a few comments that may help improve the clarity of the presentation.

1)	I appreciate that the authors used a sophisticated algorithm to directly detect many-body contacts that will form simultaneously. However, I can think of a naïve algorithm to rank the probability of the contact strength between loci i, j and k by the product of Cij and Cik. How much better is the author's algorithm compared to such naïve approaches? A comparison like this will help the reader better understand the need for developing more advanced approaches.

2)	The authors selected 39 genomic loci. A simple explanation on how they arrived at these regions would be helpful.

3)	A spherical confinement was used in polymer simulations of individual loci. While whole chromosomes are known to form territory and adopt a more or less spherical shape, individual loci of Mb in size can be quite flexible and are not necessarily spherical. The impact of biasing them with a spherical confinement needs to be better explained.

4)	The paragraph on deconvolving population Hi-C interactions into single-cell contact states needs to be refined in a way that the reader can have a rough understanding of the algorithm without studying the entire SI.

5)	The authors should use more established approaches like Fit-Hi-C to more closely examine the agreement between simulated and Hi-C contact map by focusing on statistically significant contacts.

6)	The authors claim that they can recover single-cell contact states. It then would be natural to compare such predictions with single-cell data from Xiaowei Zhuang or Sunney Xie's lab.

**Reviewer 2**

**Were you able to assess all statistics in the manuscript, including the appropriateness of statistical tests used?**

Yes: The statistical testing in the paper was appropriate for the data presented.

**Were you able to directly test the methods?**

No

**Comments to author:**

SUMMARY

Perez-Rathke et al. describe CHROMATIX, a computational method to construct 3D chromatin ensembles from widely and publically available Hi-C data to predict many-body chromatin interactions. While experiments such as SPRITE probe for many-body interactions, they do not allow for the discovery of specific interactions decoupled from random non-specific interactions that arise due to proximity effects. CHROMATIX seeks to find specific Hi-C interactions without relying heavily on the Hi-C data, avoiding biases inherent in using the data to create a background model.

CHROMATIX uses nCSAC (n-Constrained Self-Avoiding Chromatin) to simulate possible 3D conformations that exist as the null model to then generate a random Hi-C map, from which specific pairwise contacts can be selected from the actual Hi-C data. The specific contacts are coarse-grained through clustering and filtering such that 5% of the selected specific interactions are then used as a Bayesian prior to deconvolve the population average Hi-C data into interactions that could coexist in a single-cell within the geometric constraints determined by the filtered specific chromatin configurations. The deconvolved single-cell chromatin ensembles are then used to simulate Hi-C by averaging over the pairwise interactions across the population.

They find that the specific 3-body interactions are more typically involved with super-enhancers coupled with promoters relative to the non-specific complexes, which are comprised of more interactions without functional associations. For maximally higher-order many-body interactions (i.e. 4-body, 5-body, etc.), multiple super-enhancers seem to interact together as a transcriptional bridge to connect distant functional regions. Additionally, the authors used biological markers and machine learning to predict regions with specific many-body principal loops (i.e. the largest loop formed around the many-body complex); and major chromatin markers—such as DNase, RNAP2, H3K4me1, nuclear RNA, RAD21, and CTCF—result as the best classifiers for this many-body enrichment prediction.

The paper is well written and the figures are generally of high quality. The methods and supplement do a good job of explaining the work and allow it to be reproduced by others, with an exception described below. The code is nicely documented and available via bitbucket. Overall this paper will be of interest to the community of biologists that use Hi-C methods or are interested in modeling of chromosomes.

CRITIQUE

In the coarse-graining step of selected pairwise interactions, CHROMATIX uses only 5% of the configurations. It would have been nice to see the reasoning behind this, whether determined through statistics, biology, or trial-and-error. It's clear that the simulated Hi-C generated from this subset of the ensemble still reasonably recovers the pairwise Hi-C data, but it would be helpful to know the range of the minimal set of interactions needed for faithful recovery.

The comparison to SPRITE data is an important validation of the method's ability to detect many-body interactions. However, the way the SPRITE concordance is reported makes it difficult to evaluate the method against experimental data. The found fraction numbers are encouraging and the definition of the found fraction makes sense, but it is not clear how the SPRITE coverage data should be evaluated. The percentages shown in Figure S5 are very low, around 0.5% and 0.8%. Are these percentages of all SPRITE clusters or only of clusters that are covering the same genomic bin? The way the coverage is calculated should be better explained, to give the reader an idea of what percent coverage would be expected for the limit of perfect concordance with SPRITE vs. what would be expected for random data. It is also not clear why having a slightly higher coverage rate for specific vs. nonspecific contacts is indicative of good concordance with the SPRITE data. It seems like the ratio between the two does not change much with genomic distance, and that is surprising. Shouldn't non-specific contacts be more common w.r.t. specific ones at short genomic distances?

Do the many-body interactions tell us anything beyond loci being in the "A" compartment? The chromatin state analysis suggests that many of the multi-body contacts are mediated by interactions between open chromatin regions. However, it is not clear if this is due to A/B compartment segregation or more specific interactions between subsets of the A compartment. The authors should discuss this. Figure 8 is a nice illustration of the difference between 3-body and higher order interactions, but it suggests that there is a qualitative difference between them. The 4+-body interactions look like they could be caused by the "plaid" pattern of compartment interactions normally visible in Hi-C data.

Do the authors observe any interactions between polycomb-silenced regions, e.g. polycomb bodies (S. Kundu et al., Molecular Cell, 2017)?

The statement that "this work is the first to provide evidence, with measurable Euclidean distances, that super-enhancers are directly and non-randomly interacting spatially with other functional genomic regions in many-body complexes" is too strong to be supported by the data presented in this paper. The phrasing should make it a little more clear that the "measurable Euclidean distances" are estimates that need to be tested experimentally, rather than being actual measurements. Furthermore, the non-random nature of the interaction should be qualified with a discussion of how specific these contacts are beyond what would be expected for a locus in the A compartment interacting with another locus in the A compartment within a given genomic distance.

MINOR POINTS

Overall the figures are high quality, but a few points should be cleared up.

- The heat map color scheme for experimental Hi-C data is inconsistent between Fig. 1 and Fig. 2.

- The maroon and the red are hard to distinguish in Fig. 4.

- Does Figure 6b show real data or is it a cartoon? If it shows real data, units should be indicated on the axes.

- What do the linked yellow dots in Fig. S8 indicate?

Can inter-chromosomal many-body interactions be determined from this method? The modeling approach is not explained in the main paper or SI to the extent at which the reader can place definitive boundaries on the questions that CHROMATIX can and cannot answer.

**Authors Response**

**Point-by-point responses to the reviewers’ comments:**

Revision Report

We thank both referees for very helpful critique. We have followed the advice of the referees closely and have revised our manuscript extensively. The ref- erees’ thoughtful comments and suggestions have proved to be very valuable, which enabled us to strengthen our paper significantly.

Our detailed responses to the referees’ comments are itemized below.

Reviewer 1

1.	“1) I appreciate that the authors used a sophisticated algorithm to di- rectly detect many-body contacts that will form simultaneously. How- ever, I can think of a na¨ıve algorithm to rank the probability of the contact strength between loci i, j and k by the product of Cij and Cik. How much better is the author’s algorithm compared to such na¨ıve ap- proaches? A comparison like this will help the reader better understand the need for developing more advanced approaches.”

*Thank you for pointing out the need to compare our model with that of a simple naive model of a direct product of independent interactions. The difference can be illustrated in the following two aspects:*

*A.) Ours considers many scenario probabilistically, including this sim- ple naive model, which our model regress to when there are no/little dependency among the three sites of i, j, k. That is, we then have*

*π(cijk) = π(cij, cik, cjk) = π(cij ) · π(cik) · π(cjk).*

*The final model generated by the Gibbs sampler is compared to this naive model to assess if it has a higher posterior probability. This is done for all modeled loci. In fact, the hypothesis of this simple naive model is favored by default, as all other more complex models will incur a penalty term (Eqn (14) in SI).*

*Generally, we find that even after penalizing more complex models, the final models obtained through Gibbs sampling for each locus contain some form of dependency among the contact pairs. Furthermore, after evaluation using Eqn (10) in SI, these models are of higher posterior probability than the naive independence model*

*However, we do find that there is one locus (chr6:26,040,000–27,880,000) among the 39 lociu where contacts are independent. In addition, there are other genomic regions where contact interactions are mostly inde- pendent. As an example, the 1.7 MB genomic region (chr5:88,020,000– 89,700,000) has few dependencies in the final folding model: about 80% of the coarse-grained specific contacts are likely acting independently.*

*B.) This simple naive model ignores the possibility that certain com- binations of pairwise specific contacts are physically impossible. While the individual pairwise contacts may have significantly higher proba- bilities of occurrence relative to random folding, there are many in- stances where their combination is geometrically infeasible. This is reflected in the failure of our extensive polymer simulations (hundreds of CPU cores computing for weeks) in constructing 3D models of such co-occurring higher-order contacts. For the 39 loci, the mean number of likely infeasible contact combinations is 255 combinations/locus among the coarse-grained specific contacts. These infeasible combinations are used as input to our Bayesian model. Specifically, folding models (CRN - contact regulatory networks, SI section 1.3) are penalized for geomet- rically infeasible combinations (Eqn (14), SI). This process of pruning is critical for us to make reliable inferences on many-body interactions.*

*We have added the following sentences to the Methods section (Poly- mer simulation of structural perturbation):*

*We find that our models for 38 out of the 39 loci have higher pos- terior probabilities than the naive models of product of indepen- dent pair-contacts, the latter further suffers from the problem of inability of recognizing geometrically infeasible combinations of pair-contacts.*

*and towards the end of the section 1.3.3 in SI:*

*Results of applying CHROMATIX on the 39 loci studied here showed that models generated for 38 loci have higher poste- rior probabilities than a simple naive model of product of in- dependent pair-contacts. However, there may exist genomic regions where pair-contacts are largely independent. For exam- ple, we find that the 1.7 MB genomic region (chr5:88,020,000– 89,700,000) has few dependencies in the final folding model: about 80% of the coarse-grained specific contacts are likely act- ing independently.*

*and in Section 3:*

*For the 39 loci, the mean number of likely infeasible contact combinations is 255 combinations/locus among the coarse-grained specific contacts.*

2.	“2) The authors selected 39 genomic loci. A simple explanation on how they arrived at these regions would be helpful.”

*Our aim is to identify highly non-random and direct higher-order in- teractions among super-enhancers, enhancers, and promoter regions. Therefore, we decided to examine semi-insulated genomic domains with:*

*1)	a high degree of functional annotation, and 2) likely exhibiting in- teresting higher-order folding patterns among these functional regions that are currently unexplored. Hence, we choose TADs with: a) at least 2 super-enhancer (SE) and b) showed evidence of possible con- densation among these regions. For this, we focus on genomic loci from publicly available SPRITE data that contained clusters with mul- tiple co-occurring SEs. By using a minimal set of specific contacts we predicted from Hi-C, we succeeded in reconstructing 3-D structural en- sembles, which were overall consistent with both Hi-C and SPRITE data. These ensembles contain rich information beyond the original Hi-C and SPRITE data, such as explicit models of direct higher-order interactions (≤ 80 nm) among these functional regions.*

*We have added new sentences in the Introduction section*

*With the goal of studying highly non-random and direct higher- order interactions among super-enhancers, enhancers, and pro- moter regions, we apply our method to a diverse set of 39 highly transcriptionally-active loci in the GM12878 mammalian cell line; specifically, all TAD-bounded [1, 2] loci (<2 MB), each with at least 2 super-enhancers [3, 4, 5] showing evidence of pos- sible super-enhancer condensation (see Supplementary Informa- tion, Table S1) [6].*

3.	“3) A spherical confinement was used in polymer simulations of indi- vidual loci. While whole chromosomes are known to form territory and adopt a more or less spherical shape, individual loci of Mb in size can be quite flexible and are not necessarily spherical. The impact of biasing them with a spherical confinement needs to be better explained.”

*Thank you for pointing out this issue. The assumption of spherical confinement is based on the following considerations:*

*A).	TADs have relatively insular contact profiles; i.e., higher intra- domain interaction frequencies than inter-domain frequencies [1, 2]. Furthermore, effects of volume exclusion, entanglement with other chro- matin polymers, as well as nuclear organizing structures are thought to confine polymer motion into a series of “blob” like domains [7, ?], as in the classical “reptation” model. Without any other information and hence avoiding further bias, we assume that effects of nuclear connement, implicit volume exclusion from other chromatin regions, nuclear bodies, as well as components of the insular nature of TAD regions, can be reasonably captured with a model of spherical confinement.*

*The justifications of this assumption were discussed in previous works of Gu¨rsoy et al	[8].*

*B).	If individual loci are not influenced by nuclear confinement as Re- viewer 1 postulated, then our confinement model would tend to bias towards more condensed chains relative to the experimental Hi-C. This would makes our random polymer model more conservative, as random interaction frequencies would be overall increased. Therefore, it would take a more extreme Hi-C interaction frequency to be called specific. In other words, this confinement model would make our predictions more specific (higher true positive rate) but less sensitive (lower true nega- tive rate) at identifying truly significant Hi-C chromatin interactions. As a result, our model is more stringent than alternative assumptions, therefore, would give us more confidence in the chromatin interactions that were called specific. Furthermore, this level of stringency is im- portant in identifying specific many-body interactions, which is well controlled as both the random and reconstructed 3-D TAD models are under the same level of confinement.*

*We have added the following text to our manuscript (Identifying specific interactions from Hi-C data in the Results section):*

*The assumption of spherical confinement makes this null model more stringent in calling specific interactions as discussed in [8],*

*C).	Exploring the effects of different confinement indeed is an interest- ing open question. As our manuscript is a Methods paper, a detailed analysis of the effects of different confinement is beyond the focus of this study. Nevertheless, our chromatin folding tool does support modeling of different types of confinement beyond spherical, including ellipsoidal confinement, explicit nuclear lamina interaction, as well as nuclear body (e.g., nucleolar) constraints. These capabilities will be made publicly available once this manuscript is accepted. We have added the follow- ing sentences (Identifying specific interactions from Hi-C data in the Results section):*

*although our tool can be used under other confinement models such as ellipsoid.*

*and the following sentences and figure in the section	of 1.1.	Null Model of the SI:*

*The confinement of the null model can be either spherical or el- lipsoidal, both supported by the CHROMATIX implementation. An example of a chromatin polymer folded under ellipsoidal con- finement using our tool is shown below:*

4.	“4) The paragraph on deconvolving population Hi-C interactions into single-cell contact states needs to be refined in a way that the reader can have a rough understanding of the algorithm without studying the entire SI. ”

*Thank you for pointing out the confusion in our writing – indeed, this paragraph is very difficult to understand as written. We have now modified our text in Single-cell contact state deconvolution to clarify key points in our reconstruction method:*

*Once a single-cell contact state is properly generated, we then construct a set of 3D chromatin polymers that are all consistent with this single-cell contact state. By generating a large number of single-cell contact states, we can obtain an ensemble of 3D chromatin polymers, which accurately reproduce the observed population Hi-C measurements. Structural analysis of the en- semble of single-cell chromatin conformations then can reveal specific spatial many-body interactions.*

*The key to properly generating single-cell contact states is to ac- count for dependencies among chromatin interactions, namely,*

*how certain physical interactions may cooperatively induce for- mation of other interactions due to polymer folding. These dependencies are identified by in silico knock-in perturbation studies, where differential contact probabilities are assessed be- tween two ensembles of chromatin polymers, one with and an- other without the target contact knocked-in. A large number of possible dependencies are identified through these extensive polymer knock-in simulations (see Methods and Supplementary Information). Such simulations also identify geometrically infea- sible contact combinations.*

*To properly deconvolve population Hi-C interactions into single- cell contact states, we adopt a Bayesian generative approach. The dependencies and infeasible geometries among contacts are incorporated as a Bayesian prior. This physically-based prior along with the measured Hi-C data enables efficient Bayesian inference over the posterior distribution of single-cell contact states. Specifically, we use Gibbs sampling for this inference (see Supplementary Information). For efficiency, we first coarse-grain the called specific Hi-C interactions before carrying out knock-in simulations and Gibbs sampling. Only ca 5–7% specific interac- tions are retained, which substantially reduced the computation cost, making this approach highly practical.*

5.	“5) The authors should use more established approaches like Fit-Hi- C to more closely examine the agreement between simulated and Hi-C contact map by focusing on statistically significant contacts. ”

*Thank you for this suggestion. We computed the statistically significant con- tacts at each chromosome using Fit Hi-C, with a q-value significance threshold of < 1%. Among the 39 loci studied, Fit-Hi-C did not iden- tify any significant contacts for 2 loci. These 2 loci were excluded from further analysis. At the remaining 37 loci, Fit-Hi-C identified a total of 4,057 significant contacts, with a mean of 112 significant contacts per locus (standard deviation of 42 contacts/locus). We then computed the Pearson correlation between the experimental and simulated Hi-C ensembles at the (i, j) locations identified as statistically significant by Fit-Hi-C. For these loci, we obtain a mean Pearson correlation of 0.97 (standard deviation = 0.03).*

*In summary, our results show strong Pearson correlation between experimental and simulated Hi-C at contacts identified as statistically significant by Fit-Hi-C.*

*We have updated Table S1, with a new column ‘pearsonFHC’, which provides the Pearson correlation between experimental and simulated Hi-C ensembles at the contact locations identified as statistically significant by Fit-HiC. We also provide a new column ‘nFHC’ containing the number of statistically significant Fit-HiC contacts at each locus.*

*We have added the following paragraph to 7.1 Table S1 of SI:*

*Table S1 also contains information on the correlation between simulated and experimental Hi-C data at contact locations iden- tified as statistically significant by the Fit-HiC method (q < 1%). After excluding 2 loci which have no significant contact by Fit-Hi-C, the mean number of significant contacts per locus is*

*112	42. The mean Pearson correlation is	0.97	0.03. Similar results are obtained with q < 5% and q < 10%.*

*In the updated Table S1, the column ‘nFHC’containing the number of statistically significant Fit-HiC contacts at each locus; column ‘pear- sonFHC’ list the Pearson correlation at the contact locations identified by Fit-HiC. This information is now updated in the legend of Table S1.*

6.	“6) The authors claim that they can recover single-cell contact states. It then would be natural to compare such predictions with single-cell data from Xiaowei Zhuang or Sunney Xie’s lab. ”

*The work from the Zhuang lab [9] contains no GM12878 data, there- fore a direct comparison is not possible. However, following the spirit of their work, we have done a limited study (due to the limitation of revision deadline) to examine if TAD boundaries are generally consis- tent across single cells using pairwise distance matrices. Overall, we found our results are in agreement with the key findings of the Zhuang lab, which were done in K562, IMR90, and A562 cell lines.*

*We have added a new section to the Results section:*

*Reconstructed single-cell chromatin structures.*

*We have compared our single-cell chromatin models with publicly- available single-cell Dip-C data for GM12878 [10]. For each cell in the Dip-C ensemble, we identified the corresponding CHRO- MATIX cell with maximal overlap of contacts. Fig 4 shows the overall pattern of agreement and examples of individual single- cells. In general, CHROMATIX single-cell models contain more contacts (grey regions in Fig 4b-d) than that of Dip-C, but there is overall good agreement, with many long-range contacts ap- pearing in both Dip-C and CHROMATIX single cells (Fig 4b-d). The median overlap coefficient is 65% for the n = 976 cell- loci.*

*We have also added the following to the section of Comparison with single-cell studies in the SI:*

*We have obtained publicly available single-cell Dip-C data of GM12878 cells from the GEO database (GSE117874), and com- pared to our CHROMATIX cells. There are 17 Dip-C cells re- ported, two are damaged and thus removed. Each remaining Dip-C cell was then split into maternal and paternal homologs, so the Dip-C ensemble was effectively doubled to 2 15. We then examined the 39 loci in each of the 30 Dip-C cells. After removal of cells with < 10 contacts, we obtain a set of n = 976 Dip-C single-cell models for these 39 loci.*

*We calculate the overlap coefficient as [11]:*

*Overlap =	|X ∩ Y |*

*min(|X|, |Y |)*

*where X is the set of pairwise contacts within the Dip-C cell and Y is the set of pairwise contacts within the CHROMATIX cell.*

*Figure 3: Reconstructed ensemble of 25,000 single-cell chromatin structures of the locus chr X: 19,560,000 - 20,170,000 at 5 KB resolution. a) Heatmap of mean pairwise Euclidean distance in ˚A. Corresponding Hi-C heatmaps (experi- mental and simulated) can be seen in Figure S3. b) Boundary strength of mean pairwise distances computed following [9] at each 5 KB bin. c) Single-cell pair- wise distance heatmaps for two representative cells. d) Heatmap of single-cell boundary strengths, each row is the boundary strength curve of an individual cell among the 25,000 cell ensemble.*

*Figure 4: Comparison with Dip-C single-cell data (GSE117874) [10]. a.) Scatter plot of maximal overlap coefficient (Y -axis) vs number of contacts present within each Dip-C models (X-axis) of single-cell chromatin at different loci (n = 976). The horizontal boxplot shows the distribution of Dip-C contacts per cell (median 50). The vertical boxplot shows the distribution of maximal overlap coefficients between the Dip-C and CHROMATIX ensembles (median	65%). The inner and outer ellipses contain 5% and 95% of the single-cells, respectively. b, c, d). Plots of pairwise contacts between representative Dip-C cells (upper triangle, black dots) and the corresponding CHROMATIX cells (lower triangle, grey dots) of maximal overlap coefficient. Contacts present in both models are outlined in red. More details can be found in Supplementary Information.*

Reviewer 2

1.	“In the coarse-graining step of selected pairwise interactions, CHRO- MATIX uses only 5% of the configurations. It would have been nice to see the reasoning behind this, whether determined through statistics, biology, or trial-and-error. ”

*Thank you for pointing out this important issue. Indeed, the threshold of 5% does not necessarily represent the exact % of minimum set of specific interactions for reconstruct 3D ensembles consistent with Hi- C, but rather the average % retained. We have added the following sentences in the CHROMATIX 3-D Chromatin Reconstruction section of the SI:*

*The average specific contact retention fraction of 5% results from clustering with a fixed cap of at most 35 retained specific contacts per loci. This cap was chosen arbitrarily based on an initial trial run with a single locus, in which high quality reconstruction was obtained. No further attempt was made to optimize this fraction, as it affords the necessary computational cost required for large-scale perturbation simulations.*

“it would be helpful to know the range of the minimal set of interactions needed for faithful recovery.”

*We have further added the following sentences and a figure to SI to provide further information:*

*Figure 5 shows the correlation between the simulated and ex- perimental Hi-C data sets vs. the percentage of retained specific contacts among the 39 loci.*

*The Pearson correlation (orange) appears to be fairly stable, with only minimal decrease as the fraction of retained specific contacts decreases. In contrast, distance-corrected Pearson cor- relation (blue) appears to be more sensitive to a reduced specific contact retention fraction, with a steeper slope of the trend line (blue dash) compared to the original Pearson correlation trend line (orange dash).*

*Figure 5: Correlation between the simulated and experimental Hi-C data sets vs. the % of retained specific contacts among the 39 loci. The orange dots (pearson2) are the Pearson correlations after removal of the first two diagonals from the Hi-C matrix. The blue dots (pearsonDC) are the corresponding distance- corrected Pearson correlation [12]. This figure was generated from the ‘kf’, ‘pearson2’, and ‘pearsonDC’ columns provided in supplementary Table S1.*

2.	“...but it is not clear how the SPRITE coverage data should be evalu- ated. The percentages shown in Figure S5 are very low, around 0.5% and 0.8%. Are these percentages of all SPRITE clusters or only of clusters that are covering the same genomic bin? ”

*Thank you for pointing out the confusion in our write-up on SPRITE. We have added a new section of Comparison with SPRITE data to the SI to provide further explanations.*

*The SPRITE data is sparse, as it was designed for genome-wide study, which accounts for the relatively low coverage percent- ages in Figure S5. Furthermore, analysis of SPRITE data is typ- ically at a coarser resolution of 1 MB. In contrast, our analysis of Super-Enhancer, Enhancer, and Promoter higher-order struc- tures requires much finer resolution, at minimum 5 KB as in this study. However, as the SPRITE technique can capture higher- order interactions and SPRITE data for the GM12878 cell line is publicly available, it is therefore useful for us to compare our results with the SPRITE data.*

*The percentages shown in Figure S5 are over all SPRITE clusters located within the corresponding 500 KB to 2 MB TAD locus. This averages to 3,800 SPRITE clusters per locus of varying sizes ( e.g. from 3, 4, 5, ). For a 500 KB TAD at 5KB resolution, there are 100 100 possible loop anchor locations. In addition, the many-body sizes range from 3, 4, 5, 6, and beyond. When the 3,800 SPRITE clusters are distributed over*

*100 100 anchor positions and at a specific many-body size, the curse-of-dimensionality leads to overall sparse SPRITE data for analysis.*

*Although the SPRITE coverage itself is low due to this sparsity, we are able to detect statistically significant trends among specific vs. non-specific principal loops. That is, these trends, which we call SPRITE concordance measures, are quite robust in cap- turing the significant pattern of enrichment of specific principal loops relative to non-specific principal loops in the majority of loci modeled.*

*Finally, we would like to point out that we have added new results on comparison to experimental studies of chromatins in single-cells, which complement the above comparisons with the SPRITE study (please kindly see responses to Question 6 of Reviewer 1).*

“[In Figure S5] Shouldn’t non-specific contacts be more common w.r.t. specific ones at short genomic distances?”

*We apologize for this confusion in our text. We have added the following paragraphs to the new section Comparison with SPRITE data in the SI for clarification:*

*Control for genomic distance. Our model controls for genomic distance by building a separate random contact model at each j i genomic distance bin. We identify an (i, j) contact as specific based on the distribution of random contacts*

*with the same |j − i| genomic separation.*

*Therefore, an (i, j) contact that is called specific will be more common in our CHROMATIX ensemble than non-specific con- tacts with the same j i genomic distance, because we are assigning p-values based on the upper-tail of the corresponding*

*|j − i| random contact distribution. This holds true regardless of whether or not |j − i| is a short interval.*

*Since there are relatively more specific principal loops by our model at any given j i , we ask if the subset of our princi- pal loops that show up in SPRITE data also exhibit this trend. We therefore examine the coverage of principal loops of many- bodies in our model by SPRITE data. It is reasonable to expect that more specific principal loops from our model are present in*

*SPRITE data compared to corresponding non-specific principal loops at each |j − i| bin.*

“The way the coverage is calculated should be better explained, to give the reader an idea of what percent coverage would be expected for the limit of perfect concordance with SPRITE vs. what would be expected for random data. It is also not clear why having a slightly higher coverage rate for specific vs. nonspecific contacts is indicative of good concor- dance with the SPRITE data. It seems like the ratio between the two does not change much with genomic distance, and that is surprising. ”

*Thank you for this very helpful comment and suggestion. Following your advice, we have added the following paragraphs and a new figure to the SI to explain Figure S5:*

*Concordance with SPRITE. In Fig 6, the leftmost plot illustrates perfect SPRITE concordance. Here all specific principal loops (blue dots) have higher SPRITE coverage than non- specific principal loops (pink dots) at the same j i genomic distance.*

*The middle plot illustrates perfect discordance with SPRITE. Here the relationship is reversed and all specific principal loops have lower SPRITE coverage than corresponding non-specific principal loops.*

*The rightmost plot shows when the SPRITE coverage is mixed across principal loop classes. This is more reflective of real experimental data.*

*To quantify the SPRITE concordance of each of these plots, we compute the median specific SPRITE coverages at each genomic distance bin and compare to the corresponding median non- specific SPRITE coverages.*

*We then ask how likely we are to observe a given fraction of specific median coverages exceeding the corresponding non-specific median coverages, if we assume a null hypothesis where specific and non-specific labels do not matter. In other words, our null hypothesis assumes there is no relationship between our results and SPRITE data.*

*To test the null hypothesis, we randomly permute the non- specific and specific labels and recompute the fraction of specific median coverages exceeding the corresponding non-specific median coverages using the permuted labels. We repeat the permutation procedure for 1,000 times, and then we assign an upper tail p-value to the observed fraction based on this null distribution. If the observed p-value is significant under the null distribution, then we reject the null hypothesis and simply state that the data is concordant with SPRITE.*

*Figure 6: Illustration of SPRITE concordance. The trends of perfectly compat- ible vs incompatible vs mixed SPRITE coverage scenarios are shown. Here each dot is an (i, j) principal loop with: 1) x-coordinate = j i 2). y-coordinate = corresponding SPRITE coverage for principal loop (i, j). This is defined as percentage of compatible SPRITE clusters containing principal loop (i, j), where compatible clusters are those with sizes k of the k-body containing the principal loop. The color distinguishes specific vs. non-specific principal loops according to our random polymer folding model.*

3.	“Do the many-body interactions tell us anything beyond loci being in the ”A” compartment? The chromatin state analysis suggests that many of the multi-body contacts are mediated by interactions between open chromatin regions. However, it is not clear if this is due to A/B com- partment segregation or more specific interactions between subsets of the A compartment. The authors should discuss this.”

*Thank you for raising this interesting question. On average, the 39 loci modeled in this study are 90% A compartment. We have updated Table S1, so details of the proportion of A compartment and proportion of B compartment at each locus are now reported.*

*It is likely that both i) A/B segregation and ii) interactions between subsets of the A compartment contribute to the observed effects. A/B segregation is a relatively coarse-grained phenomenon, with patterns typically observed at 100 KB to 1 MB bin resolution. In contrast, our modeling is at much finer resolution (5 KB bin) and therefore al- lows insight into many-body contacts among functional regions such as Super-Enhancers, Enhancers, and Promoters. In particular, since the 39 loci modeled in this study are 90% A compartment on average, we have effectively controlled for A compartment status and, therefore, are likely capturing many sub-compartment effects (e.g., enrichment of SE, E, P many-bodies).*

*A promising area of future study would be to model B compartment regions and contrast with our current findings.*

“ Figure 8 is a nice illustration of the difference between 3-body and higher order interactions, but it suggests that there is a qualitative dif- ference between them. The 4+-body interactions look like they could be caused by the ”plaid” pattern of compartment interactions normally visible in Hi-C data.”

*Thank you for this nice observation. Indeed it is interesting that we are seeing ‘plaid’ like patterns, even at the resolution of 5 KB. However, this is fundamentally different from those arising from A/B compart- ments, which are visible as much coarser-grained properties of Hi-C data, predominantly at 100 KB to 1 MB bin resolution.*

*In contrast, our principal loop chromatin interaction patterns are oc- curring at 5 KB, namely, 1/20 to 1/200 the base-pair scale of A/B compartments. Therefore, although morphologically they appear to be similar, principal loops and A/B compartmentalization are occurring at vastly differing orders of genomic scale. Further, the genomic regions investigated in this study are predominantly A compartment ( 90% as described previously). Therefore the plaid pattern does not correspond to A/B compartmentalization. This indeed is an interesting topic for future work.*

*We have added the following sentence to the Discussion section of our main text to clarify this issue:*

*Such banding patterns at 5 KB are likely not due to A/B com- partmentalization (100 KB - 1 MB scale), as our loci are mostly (> 90%, Table S1) in A compartments.*

*Also, as previously mentioned, we have updated Table S1 with the proportion of A compartment and proportion of B compartment at each locus.*

4.	“Do the authors observe any interactions between polycomb-silenced regions, e.g. polycomb bodies (S. Kundu et al., Molecular Cell, 2017)?”

*For this study, we focused on transcriptionally active loci, so Polycomb- silenced regions were not specifically investigated. However, Polycomb group (PcG) complexes may facilitate silencing via H3K27 methylation, as indicated by Kundu et al, 2017. We did investigate the role of H3K27me3 as a predictive marker for specific principal loop formation, and an important finding is that lack of H3K27me3 was significantly predictive. This finding may be consistent with a limited role of PcG complexes in inducing principal loops within the 39 loci of this study. This result is presented in Figure 9 of the main manuscript.*

*It is our plan to investigate the role of B compartment, heterochromatic regions, and/or Polycomb-silenced regions in the future, and to compare with our current results on transcriptionally-active regions.*

5.	“ The statement that ”this work is the first to provide evidence, with measurable Euclidean distances, that super-enhancers are directly and non-randomly interacting spatially with other functional genomic re- gions in many-body complexes” is too strong to be supported by the data presented in this paper. The phrasing should make it a little more clear that the ”measurable Euclidean distances” are estimates that need to be tested experimentally, rather than being actual measurements. ”

*We have modified our statement, it now reads:*

*... this work is the first to provide computational evidence, with measurable Euclidean distances estimated from our modes, that super-enhancers are directly and non-randomly interacting spatially with other functional genomic regions in many-body complexes [6]. These predictions can be tested experimentally.*

“Furthermore, the non-random nature of the interaction should be qual- ified with a discussion of how specific these contacts are beyond what would be expected for a locus in the A compartment interacting with an- other locus in the A compartment within a given genomic distance.”

*We have added a sentence to Identifying specific interactions from Hi-C data of the Results section:*

*The assumption of spherical confinement makes this null model more stringent in calling specific interactions as discussed in [8], although our tool can be used under other confinement models such as ellipsoid.*

*and the following sentence to SI:*

*This null model of random physical chromatin chains controls effectively for genomic-distance dependent random interactions, regardless of the A/B compartmentalization of the loci.*

6.	“MINOR POINTS: - The heat map color scheme for experimental Hi-C data is inconsistent between Fig. 1 and Fig. 2.”

“- The maroon and the red are hard to distinguish in Fig. 4. ”

*We have fixed these problems. Thank you!*

“- Does Figure 6b show real data or is it a cartoon? If it shows real data, units should be indicated on the axes.”

*We apologize for this confusion. We have added the following sentence to the caption of Figure 6b:*

*Cartoon illustration of enriched vs not enriched regions.*

“- What do the linked yellow dots in Fig. S8 indicate?”

*The yellow dashed line indicates the Kolmogorov–Smirnov test statis- tic, defined as the maximum difference between the two plotted cumulative distributions, and is used to determine the corresponding p-value. We have updated the Figure S8 caption accordingly.*

“Can inter-chromosomal many-body interactions be determined from this method? The modeling approach is not explained in the main paper or SI to the extent at which the reader can place definitive boundaries on the questions that CHROMATIX can and cannot answer.”

*Yes, inter-chromosomal many-body interactions can be determined by our method. We have added the following sentence to the main text:*

*Finally, our method can simulate multiple independent loci located on separate chromosomes within the same confining nuclear volume, and can be applied to identify specific inter-chromosomal many-body interactions.*

*References*

*[1]	Elph`ege P Nora, Bryan R Lajoie, Edda G Schulz, Luca Giorgetti, Ikuhiro Okamoto, Nicolas Servant, Tristan Piolot, Nynke L van Berkum, Jo- hannes Meisig, John Sedat, et al. Spatial partitioning of the regulatory landscape of the x-inactivation centre. Nature, 485(7398):381, 2012.*

*[2]	Jesse R Dixon, Siddarth Selvaraj, Feng Yue, Audrey Kim, Yan Li, Yin Shen, Ming Hu, Jun S Liu, and Bing Ren. Topological domains in mammalian genomes identified by analysis of chromatin interactions. Nature, 485(7398):376, 2012.*

*[3]	Abraham S Weintraub, Charles H Li, Alicia V Zamudio, Alla A Sigova, Nancy M Hannett, Daniel S Day, Brian J Abraham, Malkiel A Cohen, Behnam Nabet, Dennis L Buckley, et al. Yy1 is a structural regulator of enhancer-promoter loops. Cell, 171(7):1573–1588, 2017.*

*[4]	Warren A Whyte, David A Orlando, Denes Hnisz, Brian J Abraham, Charles Y Lin, Michael H Kagey, Peter B Rahl, Tong Ihn Lee, and Richard A Young. Master transcription factors and mediator establish super-enhancers at key cell identity genes. Cell, 153(2):307–319, 2013.*

*[5]	Denes Hnisz, Brian J Abraham, Tong Ihn Lee, Ashley Lau, Vio- laine Saint-Andr´e, Alla A Sigova, Heather A Hoke, and Richard A Young. Super-enhancers in the control of cell identity and disease. Cell, 155(4):934–947, 2013.*

*[6]	Sofia A Quinodoz, Noah Ollikainen, Barbara Tabak, Ali Palla, Jan Marten Schmidt, Elizabeth Detmar, Mason M Lai, Alexander A Shishkin, Prashant Bhat, Yodai Takei, et al. Higher-order inter- chromosomal hubs shape 3d genome organization in the nucleus. Cell, 174(3):744–757, 2018.*

*[7]	Pierre-Giles de Gennes. Reptation of a polymer chain in the presence of fixed obstacles. The journal of chemical physics, 55(2):572–579, 1971.*

*[8]	Gamze Gu¨rsoy, Yun Xu, Amy L Kenter, and Jie Liang. Computational construction of 3d chromatin ensembles and prediction of functional interactions of alpha-globin locus from 5c data. Nucleic acids research, 45(20):11547–11558, 2017.*

*[9]	Bogdan Bintu, Leslie J Mateo, Jun-Han Su, Nicholas A Sinnott- Armstrong, Mirae Parker, Seon Kinrot, Kei Yamaya, Alistair N Boet- tiger, and Xiaowei Zhuang. Super-resolution chromatin tracing re- veals domains and cooperative interactions in single cells. Science, 362(6413):eaau1783, 2018.*

*[10]	Longzhi Tan, Dong Xing, Chi-Han Chang, Heng Li, and X Sunney Xie. Three-dimensional genome structures of single diploid human cells. Sci- ence, 361(6405):924–928, 2018.*

*[11]	K Vijaymeena, M. K.; Kavitha. A survey on similarity measures in text mining. Machine Learning and Applications: An International Journal, 3(1):19–28, 2016.*

*[12]	Simona Bianco, Dar´ıo G Lupia´n˜ez, Andrea M Chiariello, Carlo Annun- ziatella, Katerina Kraft, Robert Sch¨opflin, Lars Wittler, Guillaume An- drey, Martin Vingron, Ana Pombo, et al. Polymer physics predicts the effects of structural variants on chromatin architecture. Nature genetics, 50(5):662, 2018.*

**Second round of review**

**Reviewer 1**

The authors have successfully addressed my comments and I support the publication of the manuscript in its current form.

**Reviewer 2**

The authors did a good job addressing concerns expressed by both reviewers and significantly improved their paper in both clarity and content. The more detailed explanation of the method on page 4 is very good and the comparisons to Dip-C and microscopy data are important and welcome additions that make it much easier to evaluate the performance and general applicability of the method. Figures 4, especially, and Figure 5 are great additions to the paper.

Clarifications and documentation of parameter and model choices are now much better explained in the supplement, including the choice of the 5% retained specific contacts. The cartoon explaining the SPRITE concordance is helpful as well.

I support publication of this manuscript, with just a few more minor comments on figures:

Figure 5 of main text: Please label panel a with the ChrX locus coordinates to match labeling in Figure 3d, and indicate in the Figure 5 caption that Figure 3d, not all of Figure 3, is the panel to compare. Units of length are missing from the Euclidean Distance color bar label.

Figure 3 of the supplementary methods: Please explain the coloring used in the cartoon--what do the gray dotted structures represent vs. the colored solid structures? The cartoon also does not make the parent/child relationship clear in this sampling scheme.

The captions for Figure S1 and Figure S2 seem to be missing.
